# A MEG Study of Acute Arbaclofen (STX-209) Administration

**DOI:** 10.3389/fnint.2019.00069

**Published:** 2019-12-04

**Authors:** Timothy P. L. Roberts, Luke Bloy, Lisa Blaskey, Emily Kuschner, Leah Gaetz, Ayesha Anwar, Matt Ku, Marissa Dipiero, Amanda Bennett, J. Christopher Edgar

**Affiliations:** ^1^Lurie Family Foundations MEG Imaging Center, Department of Radiology, The Children’s Hospital of Philadelphia, Philadelphia, PA, United States; ^2^Center for Autism Research, Department of Pediatrics, The Children’s Hospital of Philadelphia, Philadelphia, PA, United States; ^3^Department of Pediatrics, The Children’s Hospital of Philadelphia, Philadelphia, PA, United States

**Keywords:** ASD, MEG (magnetoencephalography), arbaclofen, GABA, biomarker

## Abstract

Several electrophysiological parameters, including the auditory evoked response component M50/M100 latencies and the phase synchrony of transient and steady-state gamma-band oscillations have been implicated as atypical (to various extents) in autism spectrum disorder (ASD). Furthermore, some hypotheses suggest that an underlying neurobiological mechanism for these observations might be atypical local circuit function indexed by atypical levels of inhibitory neurotransmitter, GABA. This study was a randomized, placebo-controlled, double-blind, escalating-dose, acute investigation conducted in 25 14–18 year-old adolescents with ASD. The study assessed the sensitivity of magnetoencephalography (MEG) and MEGAPRESS “GABA” magnetic resonance spectroscopy (MRS) to monitor dose-dependent acute effects, as well as seeking to define properties of the pre-drug “baseline” electrophysiological and GABA signatures that might predict responsiveness to the GABA-B agonist, arbaclofen (STX-209). Overall, GABA levels and gamma-band oscillatory activity showed no acute changes at either low (15 mg) or high (30 mg) dose. Evoked M50 response latency measures tended to shorten (normalize), but there was heterogeneity across the group in M50 latency response, with only a subset of participants (*n* = 6) showing significant M50 latency shortening, and only at the 15 mg dose. Findings thus suggest that MEG M50 latency measures show acute effects of arbaclofen administration in select individuals, perhaps reflecting effective target engagement. Whether these subjects have a greater trend towards clinical benefit remains to be established. Finally, findings also provide preliminary support for the use of objective electrophysiological measures upon which to base inclusion for optimal enrichment of populations to be included in full-scale clinical trials of arbaclofen.

## Introduction

Although the drug arbaclofen (STX-209) is a promising candidate for pharmaceutical therapy for use in autism spectrum disorder (ASD; Veenstra-VanderWeele et al., 2017) and fragile X syndrome (Berry-Kravis et al., 2012, 2017; Henderson et al., 2012; Qin et al., 2015), unsuccessful clinical trial outcomes challenge the excitation/inhibition imbalance hypothesis of ASD (Rubenstein and Merzenich, 2003), that helped motivate the development of arbaclofen. Specifically, arbaclofen, a GABA-B agonist, was expected to restore a balance to putative excitatory-inhibitory neural circuit abnormalities in ASD and thus improve ASD symptoms. A failed clinical trial, however, is not infrequent, and in the present study we adopt the hypothesis that the phenotypic heterogeneity of ASD arises from heterogeneity in the underlying neurobiological basis. Given between-individual differences in the neurobiology of ASD, broad inclusion criteria in clinical trials, as commonly employed, would diminish the ability to resolve positive change if the drug was only effective only in a subset of participants.

To begin exploring the above, it is of interest to demonstrate, in an acute setting, whether a participant who is a potential candidate for inclusion in a clinical trial manifests evidence in support of pharmaceutical target engagement *via* a single “test” dose administration. This, however, requires an acute readout. With respect to changes in symptoms associated with a disorder, an acute readout is unlikely to be a behavioral measure (e.g., in ASD, changes in repetitive behaviors) as behavioral and symptom changes in ASD likely occur over an extended period of time (weeks to months). If achievable, however, an acute exam (or series of exams) might also provide a rational approach towards optimal dosing, without waiting weeks for behavioral changes. Furthermore, if only a subset of potential participants did exhibit an acute drug-related response, examination of this subgroup might identify candidates distinguished by demographic or other baseline characteristics.

The following report describes a single-center, randomized, placebo-controlled, double-blind, acute “biomarker” study of the pharmaceutical arbaclofen (STX-209) in 25 adolescent males with a diagnosis of ASD. The study examined the possibility that a brief and passive magnetoencephalography (MEG) electrophysiological study consisting of a pure tone auditory exam as well as a 40 Hz auditory steady-state response (ASSR) exam would demonstrate STX-209 associated changes to superior temporal gyrus auditory encoding processes in an acute ~1 h setting. Several candidate measures were assessed including the latency of a response to pure tones (M50 response, being the earliest component measurable of the auditory evoked response, although likely analogous to later components such as the M100) and the phase coherence of 40 Hz oscillatory activity, as an index of cortical circuit function. Both of these electrophysiological measures were selected to be examined in left and right primary/secondary auditory cortex given previous studies showing abnormalities in these responses in ASD and given that these auditory responses are thought to depend, in part, on the integrity of inhibitory-interneuron and pyramidal cell cortical circuits (Gandal et al., 2010; Roberts et al., 2010; Port et al., 2014; Rojas and Wilson, 2014; Edgar et al., 2015a,b). Finally, it was also hypothesized that edited magnetic resonance spectroscopy (MRS) acquired pre- and post-administration of STX-209 would reveal changes in the levels of the inhibitory neurotransmitter GABA.

## Materials and Methods

This study was approved by the local Institutional Review Board and all participants’ families gave written informed consent. When competent to do so, the adolescent participants gave verbal assent to participate.

Twenty-five adolescent males with a diagnosis of ASD were enrolled. Two subjects were excluded from analyses due to an incorrect consenting procedure. One subject withdrew from participation during the study. One other subject was screened out at neuropsychological assessment. Three participants were left-handed and one was ambidextrous.

This study was conducted “double blind.” That is, drug and placebo were identically packaged (by the supplying source) and handled by the institutional investigational drug service (IDS). For the three visits, dose was administered in two oral pills (drug was 15 mg/pill, so DD, DP, PP, where *D* = 15 mg drug and P = placebo). The IDS devised a randomization structure that was not released to the investigators until after data was acquired and analyzed. The only constraint on randomization was imposed by the FDA that, while placebo could occur 1st, 2nd or 3rd in the series, 30 mg should never precede 15 mg. As such there were three randomization options: P,15,30 or 15,P,30 or 15,30,P. Since subjects received two identical-appearing pills on each occasion they were blinded. Since the randomization scheme was not made known to the investigators until after the data analysis was complete, they too were blinded.

**Figure 1:**
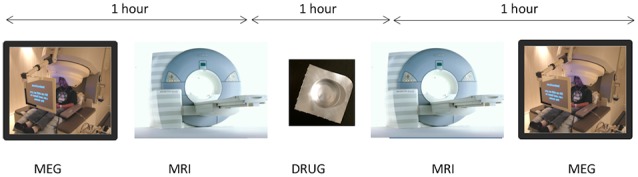
On three subsequent visits, at weekly intervals, participants underwent a protocol of baseline magnetoencephalography (MEG) followed immediately by MRI/magnetic resonance spectroscopy (MRS). Participants then received either placebo or arbaclofen at 15 mg or 30 mg dose. After approximately 1 h, MRS was repeated, followed by a MEG protocol identical to the baseline MEG exam. The entire imaging-drug-imaging process lasted approximately 3 h.

Participant demographics are shown in Table 1 and the study design depicted in Figure 1. At the first visit, a full neuropsychological evaluation was conducted including Autism Diagnostic Observation-2 (ADOS-2; Lord et al., 2012), Social Responsiveness Scale 2 (SRS-2; Constantino and Gruber, 2012), Social Communication Questionnaire (SCQ; Rutter et al., 2003) for diagnostic confirmation and the Wechsler Abbreviated Scale of Intelligence-II (Wechsler, 2011) and the Clinical Evaluation of Language Fundamentals—Fifth Edition (CELF-5) for characterization of cognitive (full scale intelligence quotient, FSIQ) and language abilities (core language standard score).

**Table 1:** Demographics.

	Age	ADOS-calibrated severity score	SRS T-score	SCQ Total score	FSIQ Standard score	CELF-5 Standard score
Sample Mean ± SD (*N* = 21)	15.8 ± 0.8 years	7.6 ± 2.1	70.1 ± 12.2	19.8 ± 7.8	75.2 ± 19.1	61.9 ± 18.2

On three subsequent visits, at weekly intervals, participants underwent a protocol of baseline MEG followed immediately by MRI/MRS. Participants then received either placebo or arbaclofen at 15 mg or 30 mg dose (in each case, two identical-appearing oral tablets). After approximately 1 h, MRS was repeated, followed by a MEG protocol identical to the baseline MEG exam. The entire imaging-drug-imaging process lasted approximately 3 h. Since the half-life of arbaclofen is reported as 4–5 h (Berry-Kravis et al., 2017), residual effects are considered unlikely after a 1-week interval.

### Paradigms and Stimuli

Two auditory exams were administered. The first auditory exam (“M50 Exam”) consisted of simple sinusoidal tones of 500 Hz frequency and 300 ms duration played binaurally at 45 dB Sensation Level (SL) corresponding to a pleasant conversational level (note SL loudness presents an equivalent sensory sensation, after determining individual hearing thresholds). Stimuli were presented through piezoelectric transducers and ear tip inserts (ER3A, Etymotic, IL, USA), with the inter-stimulus-interval (ISI) randomly varying between 600 and 2,000 ms, and with 520 trials collected over approximately 14 min. The second auditory exam (“40 Hz ASSR Exam”) consisted of a 500 Hz stimulus modulated at 40 Hz, with the modulation depth 100%. Stimuli of 1 s duration were presented with a 4 s offset-to-onset ISI (± 2 s), with 100 trials collected over approximately 17 min.

### MRI/MRS

MRI/MRS was performed on a 3T Siemens Verio MR scanner. A 3D isotropic T1-weighted structural MRI (sMRI) was acquired for the purposes of MEG source modeling. A single voxel edited MRS MEGAPRESS sequence was also administered (Mescher et al., 1998), with a voxel of 4 × 3 × 2 cm placed in the left superior temporal gyrus, and with TR/TE = 1,500/80 ms. To minimize the impact of coedited macromolecules (widely acknowledged in the conventional MEGAPRESS sequence), a modification was implemented in which the “off” pulse was delivered at 1.5 ppm frequency (symmetric about 1.7 ppm with the traditional “on” pulse at 1.9 ppm). This achieves a level of macromolecule suppression, while only extending the echo time moderately from 68 ms to 80 ms (Edden et al., 2012).

### MEG Recording and Analysis

MEG data were obtained in a magnetically shielded room using a 275-channel whole-cortex CTF magnetometer (CTF MEG, Coquitlam, BC, Canada). At the start of the session, three head-position indicator coils were attached to the scalp to provide continuous specification of the position and orientation of the MEG sensors relative to the head (Roberts et al., 2010). To minimize fatigue and encourage an awake state, subjects viewed a silent movie projected on to a screen positioned at a comfortable viewing distance. To aid in the identification of eye-blink activity, the electro-oculogram (EOG, bipolar oblique, upper right and lower left sites) was collected. To later co-register MEG and sMRI data, three anatomical landmarks (nasion and right and left preauricular points) as well as an additional 200+ points on the scalp and face were digitized for each participant using a probe position identification system (Polhemus, Colchester, VT, USA). MEG data were recorded at a sample rate of 1,200 Hz per channel using 3rd order synthetic gradiometer noise reduction and DC offset correction.

For both auditory exams, to coregister MEG and sMRI data, an affine transformation matrix that involved rotation and translation between the MEG and sMRI coordinate systems was obtained *via* a least-square match of the probe position identification points to the surface of the scalp and face. For both auditory exams, to correct for eye blinks, a typical eye blink was manually identified in the raw data (including EOG) for each participant. The pattern search function in BESA Research 6.1 (BESA GmbH, Germany) scanned the raw data to identify other blinks and computed an eye-blink average. An eye blink was modeled by its first component topography from principal component analysis (PCA), typically accounting for more than 99% of the variance in the eye-blink average. Scanning the eye blink corrected raw data, epochs with artifacts other than blinks were rejected by amplitude and gradient criteria (amplitude >300 fT, gradients >25 fT/cm).

For the pure auditory exam, non-contaminated epochs were averaged (−100 ms to 500 ms) and a 1 Hz (24 dB/octave, zero-phase) to 40 Hz (48 dB/octave, zero-phase) band-pass filter applied. Using all 275 channels of MEG data, determination of the latency of M50 sources in the left and right STG was accomplished by applying a standard source model to transform each individual’s raw MEG surface activity into brain space (MEG data co-registered to each subject’s T1-weighted 3D MRI) using a model with multiple sources (Scherg and Picton, 1991; Scherg and Ebersole, 1993; Scherg and Berg, 1996). In particular, the standard source model applied to each subject was constructed by including left and right STG dipole sources (placed at left and right Heschl’s gyrus) and the eye-blink source vector derived for each participant (Lins et al., 1993; Berg and Scherg, 1994). This source model served as a source montage for the raw MEG (Scherg and Picton, 1991; Scherg and Ebersole, 1993). As such, the MEG sensor data was transformed from channel space into brain source space where the visualized waveforms were the modeled source activities. To obtain left and right M50 latency measures, for each participant, left and right dipoles were oriented at the maximum M50 response. Thus, estimates of left and right M50 activity were obtained using an individualized anatomical constraint, with an orientation of the M50 dipoles optimized for each participant. Left and right M50 (50–125 ms) peaks were defined from the source waveforms, given appropriate magnetic field topography (ensuring the consistent orientation of neuronal current dipoles), and the latency at the left and right peak recorded.

For the ASSR exam, after artifact rejection, a band-pass filter (Butterworth) was applied with a center frequency of 40 Hz and a 20 Hz width (a band-pass filter is superior to using separate low- and high-pass filters for extracting MEG activity in narrow frequency bands) with 100% of the activity passed at 40 Hz and 50% amplitude cut offs at 30 Hz and 50 Hz. For modeling the 40 Hz steady-state response, data −500 to 1,000 ms post-stimulus were selected, with a 300 ms starting point as the amplitude-modulated 40 Hz steady-state response does not fully develop until after 250–300 ms (Ross et al., 2002). In particular, left and right STG 40 Hz steady-state dipole orientations were obtained from the 300–1,000 ms ASSR interval. Once the source model was created, the calculation of single-trial phase for the left and right STG sources used procedures outlined in Hoechstetter et al. (2004), where for each participant the derived source model was applied to the raw unfiltered data. The transformation from the time domain to the time-frequency domain used the complex demodulation technique (wavelet transformation) procedures (Papp and Ktonas, 1977) implemented in BESA 6.0, using frequencies between 4 and 60 Hz in steps of 2 Hz. Forty hertz steady-state Phase-locking (PL) was examined. PL measures were extracted from the single-trial complex time-frequency matrix. In particular, a measure of PL referred to as intertrial coherence (ITC) was computed. ITC is a normalized measure with ITC = 1 reflecting no trial-to-trial phase variability and ITC = 0 reflecting maximal phase variability across trials. For each participant, a single left and right ITC value was obtained as the average ITC within a 300–1,000 ms and 38–42 Hz interval.

### Statistics

For each dependent variable (left and right M50 latency, left and right 40 Hz ASSR ITC, and GABA/Cr), and separate for each dose, a linear mixed model (LMM) examined fixed effects of pre/post-drug, hemisphere and age, along with their interactions, and with subject as a random effect. Additionally, for each parameter, a “baseline” pre-drug/placebo standard deviation (SD) was computed from the three baseline recordings. A population SD was then estimated as the average of this baseline SD across all subjects. This was then used to recast post- vs. pre-drug (or placebo) changes in each measure as a *Z*-score (where a *Z*-score of 1 corresponds to a change in the measure of equal magnitude to the population SD). Expressing the drug/placebo-related changes in each measure as a *Z*-score provided ready comparative visualization of changes in measures that otherwise have very different units. Positive *Z*-scores represented positive changes in the measure, and negative *Z*-scores indicated negative changes. As an example, for M50 latency, a *Z*-score of “−1” is equivalent to a latency shortening of magnitude 1 population SD (approximately 5 ms). Changes were considered noteworthy when the |*Z*| score exceeded 2.57 (corresponding to a <1% probability of the change being by chance).

## Results

For placebo and for the 30 mg dose, there was no significant effect of pre- to post- placebo/drug administration on M50 latency (see Table 2). However, for the 15 mg dose, there was a significant shortening effect on M50 latency (pre: 94 ms ± 4 ms vs. post: 88 ms ± 4 ms, *p* < 0.01). *Post hoc* *t*-tests revealed an effect for the right hemisphere M50 response (pre: 97 ms ± 4 ms vs. post: 88 ms ± 4 ms, *p* = 0.012). The left hemisphere showed no significant pre- to post-difference (pre: 92 ms ± 4 ms vs. post: 87 ms ± 4 ms, *p* = 0.2). For the 40 ASSR ITC and for GABA/Cr MRS, there were no significant group effects at any dose or placebo (see Table 2).

**Table 2:** Group effects of Arbaclofen administration on MEG/MRS measures.

Placebo	Pre-administration	Post-administration	Statistics/*p*-value
M50 latency	93 ms ± 4 ms	93 ms ± 4 ms	*F*_(1,54.2)_ = 0.006, *p* = 0.941
Steady state phase locking (ITC)	0.24 ± 0.11	0.34 ± 0.12	*F*_(1,58.1)_ = 0.369, *p* = 0.55
GABA/Cr	0.151 ± 0.017	0.153 ± 0.015	*F*_(1,27)_ = 0.004, *t* = −0.065, *p* = 0.95
**15 mg dose**	Pre-administration	Post-administration	*p*-value
**M50 latency**	**94 ms ± 4 ms**	**88 ms ± 4 ms**	***F*_(1,56.0)_ = 7.48, *p* = 0.008**
Steady state phase locking (ITC)	0.28 ± 0.03	0.28 ± 0.03	*F*_(1,55.0)_ = 0.089, *p* = 0.77
GABA/Cr	0.153 ± 0.017	0.155 ± 0.019	*F*_(1,27)_ = 0.004, *t* = −0.060, *p* = 0.95
**30 mg dose**	Pre-administration	Post-administration	*p*-value
M50 latency	92 ms ± 4 ms	92 ms ± 4 ms	*F*_(1,51.5)_ = 0.021, *p* = 0.89
Steady state phase locking (ITC)	0.28 ± 0.03	0.28 ± 0.03	*F*_(1,49.7)_ = 0.138, *p* = 0.71
GABA/Cr	0.149 ± 0.014	0.163 ± 0.015	*F*_(1,30)_ = 0.526, *t* = −0.726, *p* = 0.47

*Bold face values achieve statistical significance as indicated in the far right column*.

**Figure 2:**
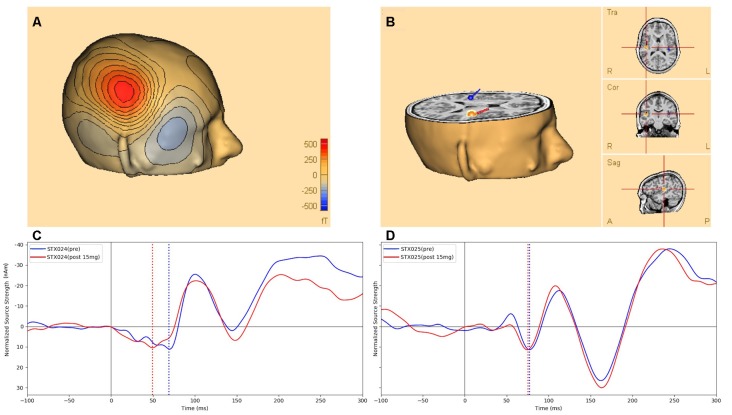
**(A)** An example of the M50 scalp magnetic field topography (over the right hemisphere) modeled by the anatomic source depicted in** (B)** and corresponding to the M50 peak deflection (dashed line at 71 ms post-stimulus) of the pre-dose source activity waveform (blue) of **(C)**, which shows the STX-209 related shortening (arrow) of the M50 latency in the modeled source waveform for a single individual pre- (blue) vs. post (red) 15 mg STX209 administration. **(D)** A corresponding example of auditory evoked waveforms from a non-responding individual pre and post a similar dose. Dotted black line marks the stimulus onset, while dotted blue (and red) lines mark the M50 response pre and post 15 mg STX209 administration. Note, by convention and for ready comparison to the ERP literature, in which negativities are shown as positive excursions from baseline and positivities are shown below the x-axis, we show the M50 response as negatively-signed and the later M100 response as positively-signed.

Figure 2A shows an example of the M50 magnetic field topography, modeled as the anatomic source(s) shown in Figure 2B at the peak M50 deflection (Figure 2C, blue dashed line). Figure 2C shows an example of STX-209-related shortening of the M50 peak latency in a single individual pre- and post-15 mg STX209, along with a representative example (Figure 2D) from a non-responding participant at the same dose.

**Figure 3:**
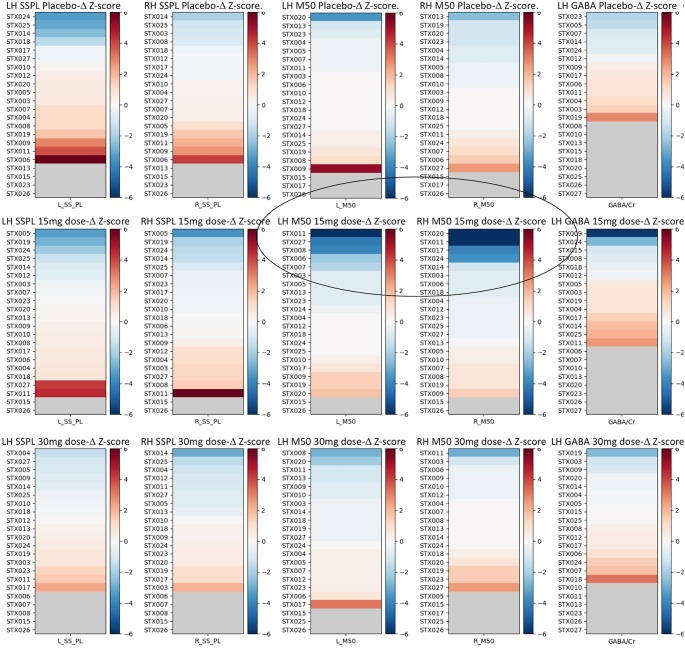
*Z*-score graphs for the imaging target variables for all participants and all measures. Interval changes post- vs. pre-administration of drug/placebo are represented in terms of *Z*-scores, where a *Z* = 1 for any measure equals a change equivalent to the population average SD of that measure across the three pre-drug/placebo baseline scans. Circled are the participants with high negative M50 latency *Z*-scores at 15 mg. The selection of responders vs. non-responders was based on a |*Z*| > 2.57 (equivalent to the 99th percentile). In each plot, subjects are identified by their subject ID (STX###) and ranked in order of their post vs. pre-effect size for each measure as a *Z*-score based on the SD derived from the three baseline scans for each measure averaged across all subjects. Hemisphere is noted as LH vs. RH. SSPL, steady state phase locking; M50, M50 latency. Increasing dependent variable values are depicted in red, and decreases in blue, with the strength of the color indicating the magnitude of the change.

Examination of the pre- to post-changes in each target parameter (bilateral M50 latency, bilateral 40 Hz ASSR ITC, and left-hemispheric GABA/Cr) revealed that the target parameters showed little change in most individuals, with occasional statistical anomalies—~1 per measure as might be expected by chance. Figure 3 shows each individual’s data at each dose, expressed as a color-coded *Z*-score.

Although most parameters showed little change as a function of STX-209 or placebo (with typically only one Z-outlier and with no systematic directional bias), the 15 mg dose appeared to have a conspicuous effect on the M50 latency. As shown in Figure 3, M50 latency *Z*-score graphs (circled), several participants had high negative M50 latency *Z*-scores at 15 mg. Furthermore, the direction of the effect (latency shortening) was the same for all participants showing an effect (i.e., there were no participants with a significant latency elongation). This observation [several Z-outliers and a directional bias (suggesting an effect not due to random chance)] motivated consideration of M50 latency as the most sensitive measurement of an acute dose-dependent effect. To this end, a subgroup of participants were defined as “M50 Responders” if their latency shortening exceeded a Z-threshold of −2.57 (equivalent to the 99% percentile). Analyses comparing six “M50 Responders” and 15 “M50 non-Responders” showed the expected finding of the “M50 Responders” having greater pre- to post- 15 mg dose M50 shortening than the “M50 non-Responders” with an interaction term of *F*_(1,57)_ = 16.02, *p* < 0.001) (see Table 3). Of note, however, 40 Hz ASSR ITC also differed between “M50 Responders” and “M50 non-Responders” (interaction term: *F*_(1,57)_ = 5.496, *p* = 0.023). There was no “M50 Responders” vs. “M50 non-Responders” group difference for GABA (interaction term: *F*_(1,25)_ = 0.061, *p* = 0.806). “M50 Responders” and “M50 non-Responders” also did not differ on any target parameter for either placebo or the 30 mg dose.

**Table 3:** Changes in target parameters with 15 mg dose, separated according to “M50 Responsiveness”.

M50 latency (ms)	Pre-administration	Post-administration	*p*-value
non-Responder	87 ms ± 4 ms	86 ms ± 4 ms	*F*_(1,57)_ = 0.143, *p* = 0.71
Responder	**112 ms ± 7 ms**	**92 ms ± 7 ms**	***F*_(1,57)_ = 25.315, *p* < 0.001**
**Steady state phase locking (ITC)**
non-Responder	0.286 ± 0.035	0.264 ± 30.035	*F*_(1,57)_ = 1.063, *p* = 0.307
Responder	**0.253 ± 0.054**	**0.325 ± 0.054**	***F*_(1,57)_ = 4.524, *p* = 0.038**
**GABA**
Non-Responder	0.159 ± 0.022	0.156 ± 0.023	*F*_(1,25)_ = 0.007, *p* = 0.934
Responder	0.140 ± 0.034	0.152 ± 0.036	*F*_(1,25)_ = 0.058, *p* = 0.812

*Bold face values achieve statistical significance as indicated in the far right column*.

Examination of the baseline parameters of the six “M50 Responders” compared to the 15 “M50 non-Responders” revealed significant baseline prolongation of M50 latency (“M50 Responders” 112 ms ± 8 ms vs. “M50 non-Responders”: 87 ms ± 5 ms, *p* < 0.05). A significant interaction between hemisphere and response status (*p* = 0.05), prompted evaluation of group baseline M50 latency differences in each hemisphere. Whereas right-hemisphere group differences were significant (“M50 Responders”: 120 ms ± 8 ms vs. “M50 non-Responders”: 87 ms ± 5 ms, *p* = 0.004), only a trend level group finding was observed in the left hemisphere (“M50 Responders”: 104 ms ± 8 ms vs. “M50 non-Responders”: 87 ms ± 5 ms, *p* = 0.09). Baseline 40 Hz ASSR ITC values did not differ between groups (“M50 Responders”: 0.157 ± −0.014 vs. “M50 non-Responders”: 0.153 ± −0.026, *p* = 0.62). Baseline GABA levels also did not differ between groups (“M50 Responders”: 0.159 ± −0.022 vs. “M50 non-Responders”: 0.140 ± −0.034, *p* = 0.65).

Examination of “M50 Responders” and “M50 non-Responders” group differences on demographic measures (two-sample *t*-test) showed no group difference in age, ADOS-CSS, SRS, SCQ, full-scale IQ, or CELF-5 core language index (Table 4).

**Table 4:** Characteristics of “M50 Responders”.

	Age (years)	ADOS-CSS	SRS	SCQ	Full Scale IQ	CELF-5
“M50 Responders”	16.2 ± 0.6	7.4 ± 2.6	73 ± 11	23 ± 11	72 ± 18	56 ± 12
“M50 non-Responders”	15.7 ± 0.9	7.3 ± 2.3	67 ± 13	19 ± 6	77 ± 20	64 ± 20
*p*-value (*t*-test)	0.20	0.96	0.33	0.37	0.58	0.26

## Discussion

Although the sample size is too small to draw strong conclusions, analyses suggested an effect of STX-209 on brain activity in only a subset of the adolescents, and only at a specific dose. In particular, 6 out of 21 adolescents (~30%) showed a significant shortening of M50 latency in response to 15 mg of arbaclofen. No other pre- to post-treatment effects were observed for any other brain measure (40 Hz ASSR or GABA) or any other dose (placebo or 30 mg). Of note, however, when the group was divided into “M50 Responders” and “M50 non-Responders,” according to their drug-related changes in M50 latency, significant STX-209 pre- to post-treatment changes were also observed for the 40 Hz ASSR ITC, with significantly higher PL *after* administration of 15 mg of STX-209. Upregulation of 40 Hz ASSR PL is consistent with the theorized mode of action of arbaclofen in a model of pyramidal interneuron network gamma (PING; Whittington et al., 2000; Jensen et al., 2014). Finally, no change in GABA level was identified at placebo, 15 mg or 30 mg dose, and GABA levels did not differ between “M50 Responders” and “M50 non-Responders.” The absence of acute response in the MRS parameter “GABA/Cr,” although counterintuitive, may, in fact, reflect the insensitivity of this measure to GABA compartmentalization or activity (on an acute timescale). While tonic GABA decrements have been reported in some cortices in ASD, it is not necessarily expected that such regionally-coarse GABA estimates (24cc) would be responsive to acute changes related to pharmaceuticals like arbaclofen.

Present findings thus suggest superior temporal gyrus M50 latency as a sensitive probe of arbaclofen activity in a subset of individuals and at a specific dose. When comparing “M50 Responder” and “M50 non-Responder,” the “M50 Responder” participants were found to have significantly longer M50 latencies at baseline (pre-drug) than the “M50 non-Responder” participants. Although this suggests that a pre-existing prolonged M50 latency may be a predictor of response to STX-209, it is important to note that some of the responsive individuals would not have been distinguished based on their baseline M50 latency alone given significant overlap between the two groups. As such, relying only on baseline M50 latency assessment and not a “test drug dose” to identify potential responders for a STX-209 clinical trial would diminish sensitivity. It is also of note that there were no baseline differences in any other MEG or MRS variable, or group differences on any of the clinical assessments of ASD severity, or cognitive or language ability. The findings highlight the utility of MEG as a modality providing exquisite temporal resolution as well as sufficient source modeling to reject surface artifact and distinguish hemispheric sources. Of note, pharmacodynamic studies using electroencephalography (EEG) either spontaneous or with stimulation as evoked potentials have been proposed for drug effect monitoring, predicting response and dose optimization for many disorders and phenomena including seizure disorders, mood disorders as well as analgesia and anesthesia—for a review, see Bewernitz and Derendorf (2012). There has, however, been less extensive work in neurodevelopmental disorders.

That the drug response in M50 latency (and also 40 Hz ASSR ITC in the subgroup of “M50 Responders”) occurred only at the 15 mg dose may suggest the need for optimal dose assessments, perhaps achieved *via* the acute dose-escalating paradigm used in this study. As the 15 mg M50 latency effect was not always observed bilaterally, this also indicates the need to examine left and right auditory activity separately. The basis for a hemisphere-specific effect in some individuals remains to be elucidated.

Two major study limitations are of note. First, although suggesting the biological activity of the drug, there is no guarantee that M50 latency responsiveness predicts a good clinical outcome in an extended clinical trial. Second, and conversely, absence of M50 latency responsiveness in a short monitoring (1 h) acute single-dose administration does not predict absence of clinical response; a single dose may be insufficient drug and a 1 h observation period may be too short.

## Conclusion

MEG measures of auditory sensory processing appear responsive to a particular dose of the GABA-B agonist, STX-209 (arbaclofen) in a subset of adolescents with ASD. It is possible that this responsiveness indicates an observable marker of differential drug biological activity in some individuals vs. others. This phenomenon could potentially be exploited as an inclusion criterion for clinical trial recruitment enrichment. Furthermore, the dose-specificity of this responsiveness could provide a mechanism for rapid determination of biologically-optimal dose. There were no observed differentiating responses in basal GABA level, estimated by MRS, either indicating the insensitivity of the MRS method or the lack of bulk GABA concentration changes associated with single-dose arbaclofen administration. Findings should be treated with caution given the small sample of responders, but indicate the possibility of observing heterogeneous responses to arbaclofen across an ASD population (possibly diminishing statistical power in a clinical trial designed to assess drug efficacy), as well as offering a tantalizing potential approach to biologically-based stratification for clinical trial enrichment and, ultimately, patient management.

## Data Availability Statement

The datasets generated for this study are available on request to the corresponding author.

## Ethics Statement

The studies involving human participants were reviewed and approved by Children’s Hospital of Philadelphia IRB. Written informed consent to participate in this study was provided by the participants’ legal guardian/next of kin.

## Author Contributions

TR, JE, LBla, AB and EK contributed to the conception and design of the study. AA and LG managed recruitment, regulatory reporting and compliance. AB was study physician. AB conducted clinical assessment. LBla and EK conducted neuropsychological assessments. MK and MD acquired the data. TR, LBlo, MK and MD performed the data analysis. TR, JE and LBlo performed the statistical analysis. TR wrote the first draft of the manuscript. JE, LBla and EK wrote sections of the manuscript. All authors contributed to manuscript revision, read and approved the submitted version.

## Conflict of Interest

TR declares his position on the advisory boards of, or consulting activity for (1) CTF MEG; (2) Ricoh; (3) Spago Nanomedicine; (4) Prism Clinical Imaging; (5) Avexis Inc.; and (6) Acadia Pharmaceuticals. TR and JE also declare intellectual property relating to the potential use of electrophysiological markers for treatment planning in clinical ASD. The remaining authors declare that the research was conducted in the absence of any commercial or financial relationships that could be construed as a potential conflict of interest.
